# Plasma phosphorylated tau 217 in preclinical Alzheimer’s disease

**DOI:** 10.1093/braincomms/fcad057

**Published:** 2023-03-06

**Authors:** Erin M Jonaitis, Shorena Janelidze, Karly A Cody, Rebecca Langhough, Lianlian Du, Nathaniel A Chin, Niklas Mattsson-Carlgren, Kirk J Hogan, Bradley T Christian, Tobey J Betthauser, Oskar Hansson, Sterling C Johnson

**Affiliations:** Wisconsin Alzheimer’s Institute, School of Medicine and Public Health, University of Wisconsin—Madison, Madison, WI 53726, USA; Wisconsin Alzheimer’s Disease Research Center, School of Medicine and Public Health, University of Wisconsin—Madison, Madison, WI 53792, USA; Department of Medicine, Division of Geriatrics and Gerontology, School of Medicine and Public Health, University of Wisconsin—Madison, Madison, WI 53792, USA; Clinical Memory Research Unit, Department of Clinical Sciences Malmö, Lund University, Lund 205 02, Sweden; Wisconsin Alzheimer’s Disease Research Center, School of Medicine and Public Health, University of Wisconsin—Madison, Madison, WI 53792, USA; Wisconsin Alzheimer’s Institute, School of Medicine and Public Health, University of Wisconsin—Madison, Madison, WI 53726, USA; Wisconsin Alzheimer’s Disease Research Center, School of Medicine and Public Health, University of Wisconsin—Madison, Madison, WI 53792, USA; Department of Medicine, Division of Geriatrics and Gerontology, School of Medicine and Public Health, University of Wisconsin—Madison, Madison, WI 53792, USA; Wisconsin Alzheimer’s Disease Research Center, School of Medicine and Public Health, University of Wisconsin—Madison, Madison, WI 53792, USA; Department of Biostatistics and Medical Informatics, School of Medicine and Public Health, University of Wisconsin—Madison, Madison, WI 53726, USA; Department of Medicine, Division of Geriatrics and Gerontology, School of Medicine and Public Health, University of Wisconsin—Madison, Madison, WI 53792, USA; Clinical Memory Research Unit, Department of Clinical Sciences Malmö, Lund University, Lund 205 02, Sweden; Department of Neurology, Skåne University Hospital, Lund 222 42, Sweden; Wallenberg Center for Molecular Medicine, Lund University, Lund 221 84, Sweden; Department of Anesthesiology, School of Medicine and Public Health, University of Wisconsin—Madison, Madison, WI 53792, USA; Department of Medical Physics, School of Medicine and Public Health, University of Wisconsin—Madison, Madison, WI 53705, USA; Department of Psychiatry, School of Medicine and Public Health, University of Wisconsin—Madison, Madison, WI 53719, USA; Wisconsin Alzheimer’s Disease Research Center, School of Medicine and Public Health, University of Wisconsin—Madison, Madison, WI 53792, USA; Department of Medicine, Division of Geriatrics and Gerontology, School of Medicine and Public Health, University of Wisconsin—Madison, Madison, WI 53792, USA; Clinical Memory Research Unit, Department of Clinical Sciences Malmö, Lund University, Lund 205 02, Sweden; Memory Clinic, Skåne University Hospital, 20502, Malmö, Sweden; Geriatric Research Education and Clinical Center of the Wm. S. Middleton Memorial Veterans Hospital, Madison, WI 53705, USA; Wisconsin Alzheimer’s Institute, School of Medicine and Public Health, University of Wisconsin—Madison, Madison, WI 53726, USA; Wisconsin Alzheimer’s Disease Research Center, School of Medicine and Public Health, University of Wisconsin—Madison, Madison, WI 53792, USA

**Keywords:** Alzheimer’s disease, amyloid beta, cognitively unimpaired, plasma, pTau_117_

## Abstract

An accurate blood test for Alzheimer’s disease that is sensitive to preclinical proteinopathy and cognitive decline has clear implications for early detection and secondary prevention. We assessed the performance of plasma phosphorylated tau 217 ($\mathrm{pTa}u_{217}$) against brain PET markers of amyloid [$\left[{}^{11}C\right]$-labelled Pittsburgh compound B (PiB)] and tau ($\left[{}^{18}F\right]$MK-6240) and its utility for predicting longitudinal cognition. Samples were analysed from a subset of participants with up to 8 years follow-up in the Wisconsin Registry for Alzheimer’s Prevention (WRAP; 2001–present; plasma 2011–present), a longitudinal cohort study of adults from midlife, enriched for parental history of Alzheimer’s disease. Participants were a convenience sample who volunteered for at least one PiB scan, had usable banked plasma and were cognitively unimpaired at first plasma collection. Study personnel who interacted with participants or samples were blind to amyloid status. We used mixed effects models and receiver–operator characteristic curves to assess concordance between plasma $\mathrm{pTa}u_{217}$ and PET biomarkers of Alzheimer’s disease and mixed effects models to understand the ability of plasma $\mathrm{pTa}u_{217}$ to predict longitudinal performance on WRAP’s preclinical Alzheimer’s cognitive composite (PACC-3). The primary analysis included 165 people (108 women; mean age = 62.9 ± 6.06; 160 still enrolled; 2 deceased; 3 discontinued). Plasma $\mathrm{pTa}u_{217}$ was strongly related to PET-based estimates of concurrent brain amyloid (${\hat{\beta }}_{}$ = 0.83 (0.75, 0.90), *P* < 0.001). Concordance was high between plasma $\mathrm{pTa}u_{217}$ and both amyloid PET (area under the curve = 0.91, specificity = 0.80, sensitivity = 0.85, positive predictive value = 0.58, negative predictive value = 0.94) and tau PET (area under the curve = 0.95, specificity = 1, sensitivity = 0.85, positive predictive value = 1, negative predictive value = 0.98). Higher baseline $\mathrm{pTa}u_{217}$ levels were associated with worse cognitive trajectories (${\hat{\beta }}_{\;pTau\times age}$ = −0.07 (−0.09, −0.06), *P* < 0.001). In a convenience sample of unimpaired adults, plasma $\mathrm{pTa}u_{217}$ levels correlate well with concurrent brain Alzheimer’s disease pathophysiology and with prospective cognitive performance. These data indicate that this marker can detect disease before clinical signs and thus may disambiguate presymptomatic Alzheimer’s disease from normal cognitive ageing.

## Graphical Abstract

**Graphical Abstract:**
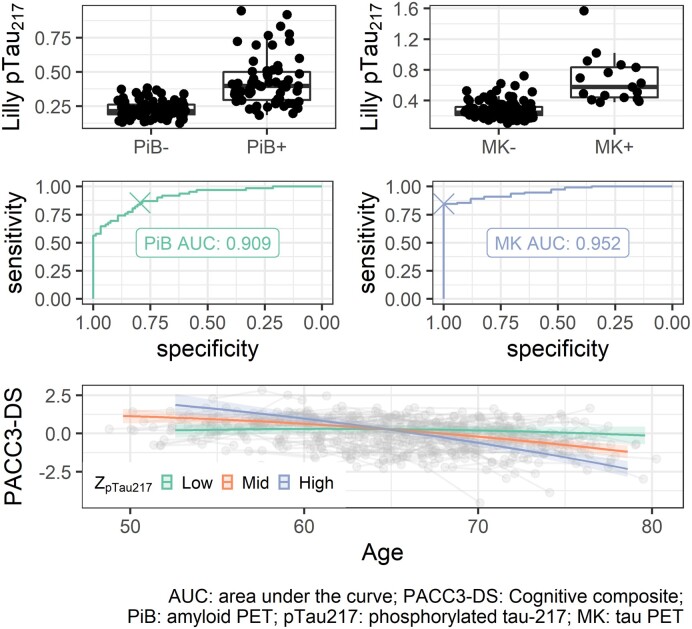

## Teaser

Jonaitis *et al*. report high correspondence between brain and plasma measures of Alzheimer’s pathology in 165 middle-aged, cognitively unimpaired adults. The area under the curves relating plasma phosphorylatedtau-217 to amyloid and tau PET positivity were 0.91 and 0.95 respectively. Results suggest promise as an early indicator of Alzheimer’s disease.

## Introduction

Blood-based biomarkers for Alzheimer’s disease that detect beta-amyloid (Aβ) and phosphorylated tau (pTau) proteinopathy are rapidly developing.^1,2^ The utility and convenience of an accurate blood test has clear implications for accelerating and improving clinical research and practice.^1–4^ Several candidate markers exist including mass spectrometry^5–7^ and immunoassay^8^ measured $A\beta_{42}$ and $A\beta_{40}$ and their ratio and phosphorylated tau at threonine 217 ($\mathrm{pTa}u_{217}$),^9^ 181 ($\mathrm{pTa}u_{181}$),^10^ and other phosphorylated sites,^11^ as well as non-specific markers of neurodegeneration and astrogliosis, including neurofilament light (NfL)^12,13^ and glial fibrillary acidic protein (GFAP).^14–16^

Recently, interest has turned to $\mathrm{pTa}u_{217}$, as cerebrospinal fluid levels increase early in autosomal dominant Alzheimer’s disease^17^ and better discriminate Alzheimer’s disease from non-Alzheimer’s subgroups of cognitively impaired adults, compared to $\mathrm{pTa}u_{181}$.^18^ In plasma, $\mathrm{pTa}u_{217}$ accurately differentiates persons with neuropathologically defined Alzheimer’s disease from other dementia.^9,19^ Further, in vivo plasma $\mathrm{pTa}u_{217}$ levels correlate with *ex vivo* protein levels and spatial burden in post-mortem brain tissue.^19–21^ Next, plasma $\mathrm{pTa}u_{217}$ levels discriminate diagnostic groups informed by amyloid PET. $\mathrm{pTa}u_{217}$ levels are elevated among impaired (Alzheimer’s disease or mild cognitive impairment (MCI)) $A\beta^{+}$ participants compared to cognitively unimpaired (CU) $A\beta^{-}$ participants,^11,19^ and plasma $\mathrm{pTa}u_{217}$ and tau PET signal show moderate to high agreement.^9,11,22^ Serial plasma $\mathrm{pTa}u_{217}$ levels also differentiate Alzheimer’s disease from non-Alzheimer’s MCI, remaining stable and non-elevated in $A\beta^{-}$ patients and increasing over time in $A\beta^{+}$ patients.^23^

The utility of plasma $\mathrm{pTa}u_{217}$ to identify amyloid and tau proteinopathy in a preclinical cohort is less well studied. Among older adults in the Swedish BioFINDER study (mean age = 72), $\mathrm{pTa}u_{217}$ levels increased over 6 years in $A\beta^{+}$ CU, but not $A\beta^{-}$,^23^ similar to findings in MCI. In this same cohort, baseline $\mathrm{pTa}u_{217}$ levels affected cognitive change.^3^ In the Australian Imaging, Biomarker & Lifestyle study (AIBL), among CU adults (mean age = 75), a 2-fold increase in levels of the $\mathrm{pTa}u_{217}+$ marker in $A\beta^{+}$ compared to $A\beta^{-}$ was recently reported,^11^ although the correlation between this biomarker and Aβ centiloids was relatively weaker in CU than that in Alzheimer’s disease and MCI, perhaps due to restriction of range (ρ = 0.64 versus 0.45). In the Mayo Clinic Study of Aging, among CU adults (mean age = 79), a smaller fold increase of 0.49 was reported in $A\beta^{+}$ compared to $A\beta^{-}$.^24^ The varying strength of these reported results may be due to measurement precision differences between instrument platforms and assays.^18,25,26^

Here we report a study from the Wisconsin Registry for Alzheimer’s Prevention (WRAP)^27^ in which we examine plasma $\mathrm{pTa}u_{217}$ trajectories in CU adults using Lilly’s immunoassay for the Meso Scale Discovery platform (Lilly-MSD).^9^ For this study, participants had a mean age of 63 at first plasma collection. We examined (i) whether changes in plasma $\mathrm{pTa}u_{217}$ levels over time track progression of Alzheimer’s disease proteinopathy ascertained from amyloid and tau positron emission tomography (PET) with $\left[{}^{11}C\right]$ -PiB for amyloid and $\left[{}^{18}F\right]$ MK-6240 for tau; (ii) whether plasma $\mathrm{pTa}u_{217}$ levels accurately differentiate people with varying degrees of amyloid and tau burden; and (iii) whether plasma $\mathrm{pTa}u_{217}$ levels are associated with longitudinal cognition.

## Methods

### Ethics

The research protocol was approved by the University of Wisconsin—Madison Health Sciences IRB (IRB00000366), and all participants provided written informed consent.

### Participants

Plasma samples were analysed from WRAP participants with ≥1 amyloid PET scan using Pittsburgh compound B (‘PiB’; see Imaging methods). Participants were included in the PiB+ sample if they had ≥ 1 global PiB distribution volume ratio (DVR) > 1.19 (centiloid equivalent = 21.6). The PiB- sample included all participants who had ≥ 2 PiB scans with all global DVR ≤ 1.1. We also examined samples from participants whose global PiB DVR trajectories indicated possible conversion from PiB- to PiB+ by virtue of initially low but recently subthreshold DVR values (1.16 < DVR ≤ 1.19). Most participants (*N* = 145) also had at least one tau scan. Primary analyses included only participants who were cognitively unimpaired (CU) at their first plasma collection and excluded one participant whose levels of $\mathrm{pTa}u_{217}$ were highly influential in models. Secondary analyses were conducted including these excluded participants (Sensitivity set 1) and excluding participants with measured $\mathrm{pTa}u_{217}$ below the lower limit of detection (Sensitivity set 2; see Plasma methods and Supplementary material).

### Plasma methods

Thirty millilitres of blood was drawn from each participant into 3×10 mL lavender top EDTA tubes (BD 366643; Franklin Lakes, New Jersey, USA). Samples were mixed gently by inverting 10–12 times and were centrifuged 15 min at 2000 g at room temperature within 1 h of collection. Plasma samples were aliquoted into 2 mL cryovials (Wheaton Cryoelite W985863; Millville, New Jersey, USA). Aliquoted plasma was frozen at −80°C within 90 min and stored for up to 10 years.

Plasma $\mathrm{pTa}u_{217}$ concentration was measured at the Clinical Memory Research Unit, Lund University (Sweden) using immunoassay on a Meso Scale Discovery (MSD) platform developed by Lilly Research Laboratories.^9^ Samples were assayed in duplicates according to published protocols^28^ with biotinylated-IBA493 used as a capture antibody and SULFO-TAG-4G10-E2 as the detector. The assay was calibrated with a synthetic $\mathrm{pTa}u_{217}$ peptide. The mean intra-assay coefficient of variation (CV) was 7.11%. The inter-assay CV for three quality control samples included in every run was 10.3%. Plasma $\mathrm{pTa}u_{217}$ concentration was below the detection limit of the assay (0.11–0.17 pg/mL) for six cases. For each model, Sensitivity analysis 2 excluded these observations. Samples were arranged on plates according to a randomization scheme devised by author E.M.J., who had no contact with samples. All samples were analysed by staff blind to clinical and imaging data.

### Imaging methods

Participants underwent T1-weighted magnetic resonance imaging as well as amyloid ($\left[{}^{11}C\right]$-PiB) and tau ($\left[{}^{18}F\right]$-MK-6240) PET imaging at the University of Wisconsin—Madison. Detailed methods for radioligand synthesis and PET and MRI acquisition, processing and quantification and analysis were implemented as reported previously.^29,30^

Amyloid burden was assessed as a global cortical average $\left[{}^{11}C\right]$ -PiB DVR, and two DVR thresholds were applied for determining PiB positivity (PiB+): one at DVR > 1.19, based on previously published work,^31^ and another, lower threshold of DVR > 1.16 (corresponding to a centiloid of 17.7), previously shown to predict subsequent accumulation of amyloid.^32^ Estimated age of amyloid onset (EAOA) was obtained from observed global PiB DVR using a combination of group-based trajectory modelling and Bayes’ theorem^33^ using either the most recent PET scan (for those who were PiB-) or the scan closest to the PiB+ threshold. Amyloid duration was then estimated as age at plasma sample minus EAOA, and the corresponding estimated PiB DVR (GBTM-DVR) was calculated via linear transformation as described in Betthauser *et al*.^34^ Centiloids were estimated from these DVRs according to the following equation: CL=148.33×DVR−154.96.

The $\left[{}^{18}F\right]$-MK-6240 standardized uptake volume ratio (SUVR) (70–90 min; cerebellum grey reference region excluding the superior medial vermis) tau burden was assessed visually by an expert reader (SCJ) using SUVR images overlaid on the coregistered MRI and scaled from 0 to 2.5. Images were classified as tau negative or tau positive for the medial temporal lobe (MTL; entorhinal cortex, amygdala or hippocampus) and for the neocortex (Neo; 1 or more cortical regions). The visual rating defined four classes: MK-, MK+ in MTL only, MK+ in neocortex only and MK+ in MTL and neocortex.

### Neuropsychological assessment and cognitive status

Participants in WRAP completed a comprehensive cognitive battery at each visit, including tests of memory, executive function, language ability and other aspects of cognitive performance, alongside self- and informant-based measures of everyday functioning.^27^ Based on these measures, participant cognitive status at each visit was determined via consensus conference.^35^ Among those without clinically significant cognitive impairment (i.e. dementia or MCI), some were assigned a research diagnosis of ‘cognitively unimpaired-declining’ denoting performance within the range of normal, but suggestive of decline from baseline.^36^

Our measure of global cognition was a three-test version of the Preclinical Alzheimer’s Cognitive Composite^37^ including the Rey Auditory Verbal Learning Test, sum of Trials 1–5; the Wechsler Memory Scale Logical Memory II, total score; and the Wechsler Adult Intelligence Scale-Revised Digit Symbol Substitution, total score. The tests were combined by rescaling and computing an unweighted average, scaled such that first observations in cognitively unimpaired indiduals were distributed ∼N(0,1). The Wide-Range Achievement Test Reading Subtest (standard score) was used as a measure of literacy.

### Statistical analysis

Statistical analyses were performed in R 4.0.5.^38^ Longitudinal $\mathrm{pTa}u_{217}$ trajectories were modelled using mixed effects models^39^ with participant-level random intercepts, which are robust to missingness when data are missing at random. Two such models were fit. First, to evaluate how well $\mathrm{pTa}u_{217}$ measurements reflect brain amyloid, we estimated the fixed effect of GBTM-DVR at each timepoint. Second, to compare age trends in people known to be accumulating amyloid versus people who are not,^23^ we estimated the fixed effects of age, amyloid status (PiB+ versus PiB-) and their interaction. An exploratory analysis estimated whether this relationship was moderated by education. Effect sizes were estimated by $\omega^{2}$,^40^ where $0.01\leq \omega^{2}<0.06$ was considered a small effect size, $0.06\leq \omega^{2}<0.14$ medium and $\omega^{2}\geq 0.14$ large.^41^ Test–retest reliability was assessed using the intraclass correlation. Significance tests were evaluated using α = 0.05.

To establish potential thresholds with maximal correspondence between $\mathrm{pTa}u_{217}$ and binary brain amyloid (global PiB) and tau (MTL+ neocortical MK-6240) positivity, receiver–operator characteristic (ROC) curves^42^ were constructed on a sub-sample comprising one plasma observation per participant, acquired within 2 years of a PiB or MK-6240 scan, respectively. Thresholds were selected to maximize Youden’s index.^43^ Positive and negative predictive values for PiB- and MK-positivity assumed population prevalence of 25% and 10%, respectively. In a secondary analysis, we used a robust norms approach to identify an alternate threshold for pTau217 by first winnowing the sample to solidly PiB- individuals (DVR < 1.1 at all scans); computing the 2.5th and 97.5th percentiles; selecting all observations within this range; and recomputing the 97.5th percentile to obtain the robust norms threshold. To validate these thresholds, we classified GBTM-DVR at each plasma observation into PiB- (GBTM-DVR ≤ 1.19) and PiB+ (GBTM-DVR > 1.19) and compared this against positivity on $\mathrm{pTa}u_{217}$ according to each threshold.

To evaluate the relationship between baseline $\mathrm{pTa}u_{217}$ and cognitive trajectories, we fit a mixed effects model of longitudinal PACC-3, with linear and quadratic age terms and their interaction with baseline $\mathrm{pTa}u_{217}$ modelled as continuous fixed effects, and a participant-level random intercept. Age and $\mathrm{pTa}u_{217}$ terms were mean-centred. Sex, education, baseline literacy and number of prior exposures to the cognitive battery were included as covariates. For comparison, a covariate-only model was also fit. An exploratory analysis estimated whether this relationship was moderated by education.

To assess whether within-person change in $\mathrm{pTa}u_{217}$ predicts within-person change in cognition and explore the phasing of this relationship, an exploratory, repeated measures correlation analysis was performed.^44,45^ This analysis used a subset of data in which $\mathrm{pTa}u_{217}$ was paired variously with concurrent PACC-3 scores and with PACC-3 lagged by one or two study visits. Only participants with at least four timepoints were included (*N* = 46; $N_{\mathrm{obs}}$ = 93), to satisfy the constraints that each participant should contribute at least two $\mathrm{pTa}u_{217}$ observations, to assess within-person change, and that each such observation should allow for pairings with cognition under three lag conditions [e.g. $\mathrm{pTa}u_{217}$ at (A) Visits 1 and 2 with PACC-3, (B) Visits 2 and 3 and (C) Visits 3 and 4, in successive models lag = 0, lag = 1, lag = 2]. This exploratory analysis was repeated twice, first using PACC-3 and $\mathrm{pTa}u_{217}$ values from which age had been partialed out and then substituting GBTM-DVR for $\mathrm{pTa}u_{217}$.

### Data availability

Coded data may be shared at the request of any qualified investigator. R scripts underlying all analyses are available in the Supplementary material.

## Results

### Participants

A total of 173 participants had qualifying PiB scans and at least 1 plasma sample stored in EDTA. At their last PiB scan, 74 had PiB DVR > 1.19, and 99 had PiB DVR ≤ 1.19. Included in the second set were 84 having ≥ 2 PiB scans with all DVR ≤ 1.11 and 15 with values suggestive of possible conversion. From this set, eight participants were removed from primary analyses due to cognitive impairment at first plasma (*N* = 6), missing diagnosis at first plasma (*N* = 1) or measured $\mathrm{pTa}u_{217}$ levels found to be highly influential using Cook’s *d* (*N* = 1). Participant characteristics for the primary analysis sample are shown in Table 1. For each applicable aim, excluded participants were included in a sensitivity analysis (Sensitivity set 1; see Supplementary Table 1 and Supplementary Figs 1 and 3). Six observations had measured $\mathrm{pTa}u_{217}$ values below the lower limit of detection; these were excluded for a second sensitivity analysis (Sensitivity set 2; see Supplementary Table 1 and Supplementary Figs 2 and 4).

**Table 1:** Demographics and background characteristics of primary analysis sample

Variable	Value
Number of participants	165
Number of plasma observations per participant, median (range)	3 (1–5)
Age at first plasma, mean (SD)	62.94 (6.06)
Age at last plasma, mean (SD)	68.22 (6.10)
Years of plasma follow-up, mean (SD)	5.28 (1.62)
Female, *N* (%)	108 (65%)
Male, *N* (%)	57 (35%)
White, *N* (%)	158 (96%)
Black, *N* (%)	4 (2%)
Native American, *N* (%)	3 (2%)
e2/e3, *N* (%)	9 (5%)
e2/e4, *N* (%)	7 (4%)
e3/e3, *N* (%)	77 (47%)
e3/e4, *N* (%)	63 (38%)
e4/e4, *N* (%)	9 (5%)
Cognitively unimpaired-stable at first plasma, *N* (%)	131 (79%)
Cognitively unimpaired-declining at first plasma, *N* (%)	34 (21%)
Cognitively unimpaired-stable at last plasma, *N* (%)	134 (81%)
Cognitively unimpaired-declining at last plasma, *N* (%)	23 (14%)
MCI at last plasma, *N* (%)	7 (4%)
Dementia at last plasma, *N* (%)	1 (1%)
PiB- at last plasma, *N* (%)	95 (58%)
PiB+ at last plasma, *N* (%)	70 (42%)
MK- at last plasma, *N* (%)	111 (67%)
MK+ MTL only at last plasma, *N* (%)	6 (4%)
MK+ Neo only at last plasma, *N* (%)	2 (1%)
MK+ MTL+ Neo at last plasma, *N* (%)	26 (16%)
MK+ missing, *N* (%)	20 (12%)

MCI = mild cognitive impairment; MK = $\left[{}^{18}F\right]$ -MK-6240 tau tracer; MTL = medial temporal lobe; Neo = neocortex; PiB = Pittsburgh compound B.

### Longitudinal $\mathrm{pTa}u_{217}$ trajectories

**Figure 1:**
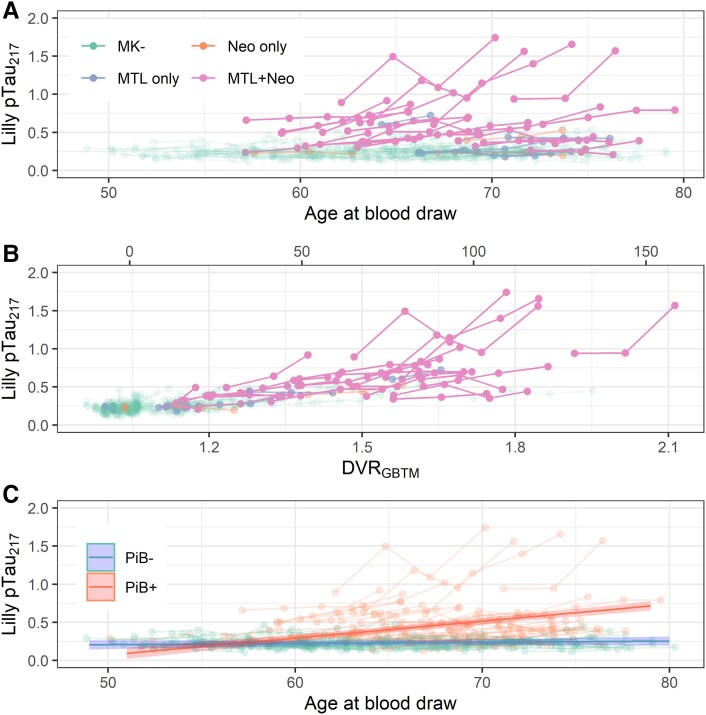
**Longitudinal plasma $pTau_{217}$. Observations from a single participant are shown with connected edges.** (**A**) Plasma $\mathrm{pTa}u_{217}$ as a function of age at blood draw. Colour indicates the extent of tau burden for each participant as indicated on tau PET (MK- = no tau signal; MTL only = tau signal in medial temporal lobe only; Neo only = tau signal in neocortex only; MTL+ Neo = tau signal in both medial temporal lobe and neocortex). (**B**) Plasma $\mathrm{pTa}u_{217}$ as a function of estimated PiB DVR at the time of plasma acquisition ($\mathrm{DV}R_{\mathrm{GBTM}}$). Colour indicates the extent of tau burden as indicated on tau PET. (**C**) Plasma $\mathrm{pTa}u_{217}$ as a function of age at blood draw. Colour indicates amyloid PET positivity. Lines with shaded confidence bands represent slope estimates from a linear mixed effects model of $\mathrm{pTa}u_{217}$ as a function of the interaction of age and amyloid positivity, the results of which were reported in Table 2B (*t*(508.84) = 7.96, *P* = 0.000000000000011). DVR = distribution volume ratio; GBTM-DVR = group-based trajectory modelled DVR from amyloid PET; PiB = Pittsburgh compound B; $\mathrm{pTa}u_{217}$ = phosphorylated tau 217.

Individual $\mathrm{pTa}u_{217}$ trajectories are displayed by age in Fig. 1A and by GBTM-DVR in Fig. 1B (secondary *x*-axis: centiloid conversions, at top). A strong relationship with MK-6240 PET is evident: those with tau in both medial temporal and neocortical regions appear distinct from other groups. The improved alignment in Fig. 1B supports strong correspondence between plasma $\mathrm{pTa}u_{217}$ and PiB PET.

The mixed effects models relating $\mathrm{pTa}u_{217}$ levels to GBTM-DVR are shown in Table 2. GBTM-DVR was a strong predictor of measured $\mathrm{pTa}u_{217}$ (${\hat{\beta }}_{\mathrm{DVR}}$ = 0.83, $\omega^{2}$ = 0.64). Intraclass correlations were moderate. Together, the results indicate good test–retest reliability and high sensitivity to true underlying change. The results of sensitivity analyses were substantially similar.

**Table 2:** The results of linear mixed effect models of $pTau_{217}$. Each model included a per-participant random intercept. For each, Sensitivity analysis 1 included observations from eight additional participants who were either cognitively unimpaired or missing a cognitive diagnosis at first available plasma draw (*N* = 7) or whose $pTau_{217}$ values were highly influential (*N* = 1), and Sensitivity analysis 2 excluded six single observations on five participants for which measured $pTau_{217}$ values fell below the lower limit of detection

$\mathrm{pTa}u_{217}$ as a function of GBTM-DVR									
	Primary set			Sensitivity set 1			Sensitivity set 2		
*Predictors*	*Estimates*	*CI*	*P*	*Estimates*	*CI*	*P*	*Estimates*	*CI*	*P*
Intercept	0.00	−0.08–0.08	0.968	0.00	−0.12–0.12	0.972	0.00	−0.08–0.08	0.984
GBTM-DVR	0.83	0.75–0.90	<0.001	0.46	0.35–0.56	<0.001	0.83	0.75–0.90	<0.001
**Random effects**									
*σ^2^*	0.12			0.32			0.12		
*τ*_00_	0.25 _Reggieid_			0.52 _Reggieid_			0.25 _Reggieid_		
ICC	0.67			0.62			0.67		
*N*	165 _Reggieid_			173 _Reggieid_			165 _Reggieid_		
Observations	515			530			509		
Marginal *R^2^*/conditional *R^2^*	0.648/0.885			0.200/0.695			0.649/0.885		
$\mathrm{pTa}u_{217}$ **as a function of age, moderated by binary PiB status**									
Intercept	0.23	0.20–0.27	<0.001	0.27	0.21–0.34	<0.001	0.23	0.20–0.27	<0.001
Amyloid positivity	0.19	0.13–0.24	<0.001	0.16	0.06–0.26	0.002	0.19	0.13–0.24	<0.001
Age (centred), linear	0.00	−0.00–0.00	0.337	0.00	−0.00–0.01	0.267	0.00	−0.00–0.00	0.350
Amyloid positivity × age (centred), linear	0.02	0.02–0.03	<0.001	0.01	0.00–0.03	0.021	0.02	0.02–0.03	<0.001
**Random effects**									
*σ^2^*	0.01		0.05				0.01		
*τ*_00_	0.03 _Reggieid_		0.09 _Reggieid_				0.03 _Reggieid_		
ICC	0.81		0.66				0.81		
*N*	165 _Reggieid_		173 _Reggieid_				165 _Reggieid_		
Observations	515		530				509		
Marginal *R^2^*/conditional *R^2^*	0.355/0.879		0.093/0.695				0.356/0.878		

GBTM-DVR = group-based trajectory modelled distribution volume ratio from amyloid PET; ICC = intraclass correlation; $\mathrm{pTa}u_{217}$ = phosphorylated tau 217.

Bold text denotes statistical significance.

The mixed effects models relating $\mathrm{pTa}u_{217}$ levels to age, with binary PiB status (PiB DVR > 1.19) as a moderator, are shown in Table 2. A mid-sized, significant age by amyloid status interaction was observed such that levels of $\mathrm{pTa}u_{217}$ increased with age only in PiB+ participants, whereas in PiB- participants, the age-related slope estimate was indistinguishable from zero (${\hat{\beta }}_{\mathrm{PiB}\times \mathrm{age}}$ = 0.021; ${\hat{\beta }}_{\mathrm{PiB}\text{-}}$ = 0.0016, ${\hat{\beta }}_{\mathrm{PiB}\;+}$ = 0.022; $\omega^{2}$ = 0.11). One participant with high $\mathrm{pTa}u_{217}$ levels continued to be highly influential in this model. The model fit to the primary dataset is shown in Fig. 1C. The results of sensitivity analyses were similar. An exploratory analysis indicated that education was not a moderator of this effect (${\hat{\beta }}_{\mathrm{ed}\times \mathrm{PiB}\times \mathrm{age}}$ = −0.00082, $\omega^{2}$ = 0; see Supplementary Table 1).

### $\mathrm{pTa}u_{217}$ threshold estimation

**Figure 2:**
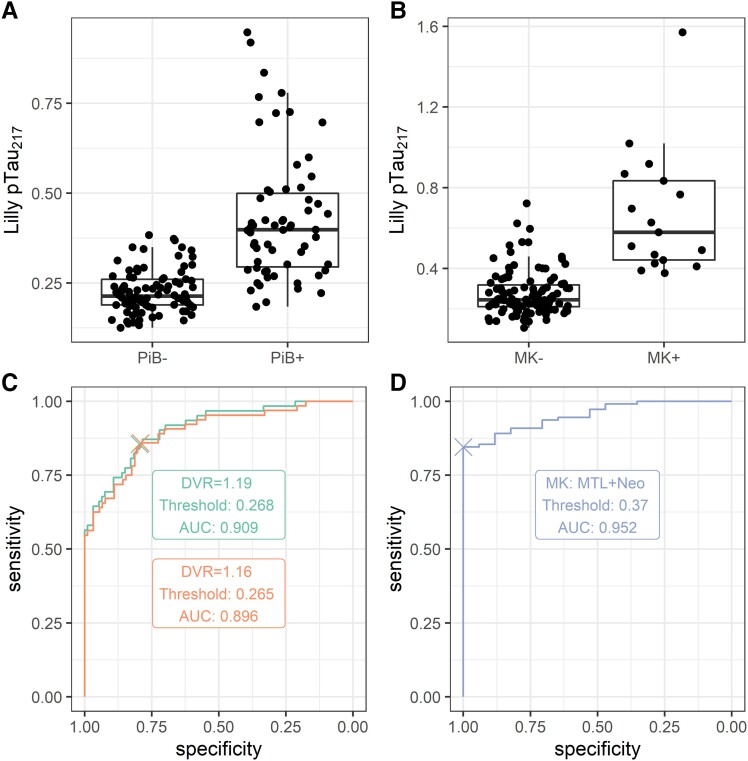
**Relationship between $pTau_{217}$ and PET Alzheimer’s disease biomarkers.** (**A**) Distribution of $\mathrm{pTa}u_{217}$ among PiB- and PiB+ participants. (**B**) Distribution of $\mathrm{pTa}u_{217}$ among MK- and MK+ participants. (**C**) ROC curve relating $\mathrm{pTa}u_{217}$ to binary PiB status. Two positivity thresholds were considered for PiB: global DVR > 1.19 (red) and global DVR > 1.16 (blue). (**D**) ROC curve relating $\mathrm{pTa}u_{217}$ to binary MK status. Scans were marked as MK+ if tracer binding was evident in both medial temporal lobe and neocortex, and MK- otherwise. DVR = distribution volume ratio; MK = $\left[{}^{18}F\right]$ -MK-6240 tau tracer; PiB = Pittsburgh compound B; $\mathrm{pTa}u_{217}$ = phosphorylated tau 217; ROC = receiver–operator characteristic.

Boxplots and ROC curves relating $\mathrm{pTa}u_{217}$ to binary PiB and MTL+ neocortical MK-6240 are shown in Fig. 2A–D. Correspondence was high for both PiB positivity thresholds [early positivity (DVR > 1.16), AUC = 0.90, PPV = 0.58, NPV = 0.94; late positivity (DVR > 1.19), AUC = 0.91, PPV = 0.58, NPV = 0.94], as well as for MTL+ neocortical MK-6240 positivity (AUC = 0.95, PPV = 1.00, NPV = 0.98). Estimated thresholds were lower for amyloid than tau (early PiB positivity: 0.27; late PiB positivity: 0.27; MTL+ neocortical MK-6240 positivity: 0.37). Our robust norms threshold approach identified a higher $\mathrm{pTa}u_{217}$ positivity boundary of 0.37. Analyses on the sensitivity datasets were similar (see Supplementary Figs 1 and 2).

When adjudicating both the late PiB positivity, ROC-based threshold and the robust norms threshold against the ground truth of concurrent GBTM-DVR > 1.19, the ROC-based threshold was more sensitive, but less specific (ROC threshold: sensitivity = 0.91, specificity = 0.75; robust norms threshold: sensitivity = 0.7, specificity = 0.96). Among individuals having ≥1 plasma $\mathrm{pTa}u_{217}$ observation between these thresholds (*N* = 70), half were PiB+ at their last PET scan (*N* = 36).

### Associations with longitudinal cognition

**Figure 3:**
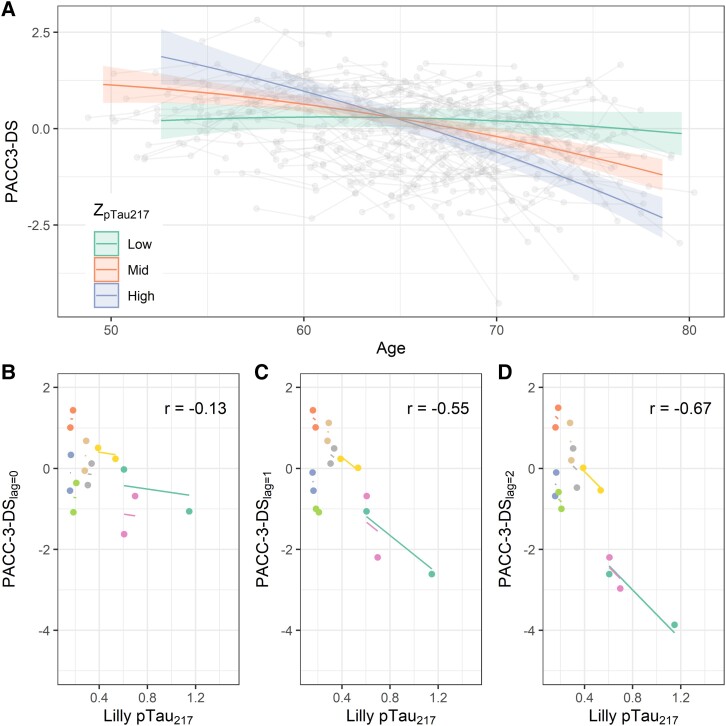
**Relationships between $pTau_{217}$ and longitudinal cognition.** (**A**) Global cognition (PACC-3) as a function of age and baseline $\mathrm{pTa}u_{217}$ level. Individual observations from a single participant are connected by grey line segments. Superimposed coloured lines reflect estimated simple main effects of age from the mixed effects model reported in Table 3, Model 2 (*t*(493.43)=−7.58, *P* = 0.00000000000017). Low, medium and high $\mathrm{pTa}u_{217}$ values reflect the 10th, 50th and 90th sample percentiles. (**B**–**D**) Repeated measures correlations (*r*; *df* = 46) between global cognition (PACC-3) and $\mathrm{pTa}u_{217}$ with lags of 0 (**B**; *P* = 0.37), 1 (**C**; *P* = 0.000059) and 2 (**D**; *P* = 0.00000020) visits between biomarker and cognitive test. The steeper slope in the rightmost panel suggests a stronger negative relationship between earlier $\mathrm{pTa}u_{217}$ and downstream PACC-3. PACC-3: three-test preclinical Alzheimer’s cognitive composite; $\mathrm{pTa}u_{217}$ = phosphorylated tau 217.

Figure 3A illustrates the mixed effects model relating baseline $\mathrm{pTa}u_{217}$ levels to PACC-3 trajectories. We observed a mid-sized, significant age by $\mathrm{pTa}u_{217}$ interaction (${\hat{\beta }}_{\mathrm{pTau}217\times \mathrm{age}}$ = −0.075, $\omega^{2}$ = 0.10). Lower baseline $\mathrm{pTa}u_{217}$ levels were associated with a flatter cognitive trajectory, whereas moderate and higher levels were linked to faster decline. To facilitate deeper understanding of the model output, we used the betas from the sex main effect, the age terms and the $\mathrm{pTa}u_{217}\times \mathrm{age}$ interaction to estimate the ages at which men and women at the 10th and 90th $\mathrm{pTa}u_{217}$ percentiles (but with otherwise average values on the other model terms) would be expected to decline to a PACC-3 score of *z* = −1.5. According to this model, the predicted age for an average woman at the 10th $\mathrm{pTa}u_{217}$ percentile to reach this *z*-score is 91.5, compared with age 74.2 for an otherwise similar woman at the 90th $\mathrm{pTa}u_{217}$ percentile. For men, these estimated ages are 86.4 and 71.7, respectively. A likelihood ratio test comparing this model to a covariate-only version indicated better performance when including $\mathrm{pTa}u_{217}$ ($\chi^{2}(3)$ = 70.7, *P* < 0.0001). Model summaries for these two models are shown in Table 3. Sensitivity analyses were similar (see Supplementary Table 2 and Supplementary Figs 3 and 4). An exploratory model adding higher-level interactions with education was not a better fit ($\chi^{2}(5)$ = 5.9, *P* = 0.32), and three-way interactions with age were small and not significant ($\omega^{2}$ = 0; see Supplementary Table 3).

**Table 3:** The results of linear mixed effect models of PACC-3 as a function of (i) covariates only (sex, education, baseline literacy and age), (ii) covariates plus baseline $pTau_{217}$ and its interaction with age. In both models, age was modelled as a second-degree polynomial. Models included a per-participant random intercept

	Base model			With pTau217		
*Predictors*	*Estimates*	*CI*	*P*	*Estimates*	*CI*	*P*
Intercept	−3.71	−5.43–−1.99	<0.001	−4.14	−5.83–−2.44	<0.001
Sex (male)	−0.41	−0.69–−0.13	0.004	−0.49	−0.77–−0.22	0.001
Education (years)	0.09	0.01–0.16	0.024	0.10	0.03–0.17	0.007
Baseline literacy	0.02	0.00–0.04	0.026	0.02	0.00–0.04	0.015
Practice	0.12	0.05–0.19	0.001	0.14	0.07–0.21	<0.001
Age (centred), linear	−0.07	−0.10–−0.05	<0.001	−0.09	−0.11–−0.06	<0.001
Age (centred), quadratic	−0.00	−0.00–−0.00	0.001	−0.00	−0.00–−0.00	0.015
Baseline pTau217				−0.07	−0.25–0.11	0.466
Baseline pTau217×age [centred], linear				−0.07	−0.09–−0.06	<0.001
Baseline pTau217 × Age (centred), quadratic				−0.00	−0.00–0.00	0.666
**Random effects**						
*σ^2^*	0.24			0.20		
*τ*_00_	0.64 _Reggieid_			0.63 _Reggieid_		
ICC	0.73			0.76		
*N*	165 _Reggieid_			165 _Reggieid_		
Observations	509			509		
Marginal *R^2^*/conditional *R^2^*	0.237/0.793			0.330/0.840		

ICC = intraclass correlation; $\mathrm{pTa}u_{217}$ = phosphorylated tau 217.

Bold text denotes statistical significance.

Exploratory lagged repeated measures correlations on a subset of individuals with at least four observations (*N* = 46) suggested weak within-person correspondence between $\mathrm{pTa}u_{217}$ and concurrent cognition ($r_{lag=0}$ = −0.130) but stronger within-person relationships when a lag of one ($r_{lag=1}$ = −0.550) or two visits ($r_{lag=2}$ = −0.670; Fig. 3B–D) was imposed. This pattern held when repeated measures correlation was performed instead after age had been partialed out from both $\mathrm{pTa}u_{217}$ and cognitive scores ($r_{lag=0}$ = −0.0720; $r_{lag=1}$ = −0.500; $r_{lag=2}$ = −0.630). In comparison, relationships with modelled PiB DVR at the same lags were weaker ($r_{gbtm.lag=0}$ = −0.0230; $r_{gbtm.lag=1}$ = −0.410; $r_{gbtm.lag=2}$ = −0.540).

## Discussion

We characterized the temporal dynamics of Lilly-MSD $\mathrm{pTa}u_{217}$ in a cohort of late middle-aged adults without baseline clinical cognitive impairment at a mean age of 63. We observed a strong relationship between brain amyloid positivity and $\mathrm{pTa}u_{217}$, with plasma biomarker trajectories rising with age only in PiB+ individuals. This is similar to a recent report from BioFINDER^23^ in a sample approximately 10 years older. We further observed strong relationships between $\mathrm{pTa}u_{217}$ trajectories and brain tau as measured via MK-6240: those with extensive tau deposition, all PiB+, exhibited increasing plasma levels of $\mathrm{pTa}u_{217}$. This resembles recent findings in AIBL of a moderately strong cross-sectional correlation between $\mathrm{pTa}u_{217}+$ and meta-temporal and mesial temporal MK-6240 SUVR in A+ older adults.^11^ However, in that analysis, the correlation was weaker in a subset of CU participants, whereas in the present analysis, which includes only CU individuals and again features a cohort 10 years younger, the relationship is strong.

Plasma biomarkers have potential for prescreening Alzheimer’s disease biomarker–positive participants in clinical trials.^3^ To that end, our results are encouraging, as we observed strong relationships between plasma $\mathrm{pTa}u_{217}$ and concurrent brain imaging biomarkers of Alzheimer’s disease, with an AUC of approximately 0.91 for identifying PiB+ participants and 0.95 for identifying those who were MK+. These values are similar to those seen for the easier task of discriminating Alzheimer’s disease $A\beta^{+}$ from CU $A\beta^{-}$ groups and are high compared to other reports describing cognitively unimpaired elderly groups in AIBL,^11^ MCSA^24^ and BioFINDER.^9^ With our threshold for predicting PiB+, the PPV of $\mathrm{pTa}u_{217}$ was 0.58, which would reduce the number needed to screen to obtain a full sample. However, for other purposes, a more conservative threshold might be preferable. For MK, in contrast, the PPV of 1 and NPV of 0.98 are likely overestimates but suggest this threshold may work well for many purposes, in principle, in populations with prevalence close to our estimates.

Our two analyses relating baseline $\mathrm{pTa}u_{217}$ to PACC-3 scores were complementary, each suggesting important longitudinal relationships between this plasma biomarker and cognition. In our primary analysis, the $\mathrm{pTa}u_{217}$ by age interaction suggests those with higher baseline biomarker levels evince worse cognitive trajectories with age than do those with lower biomarker levels, whose cognitive trajectories appear flat. In our exploratory analysis using repeated measures correlation, although the within-person relationship between biomarker levels and concurrent cognitive performance is weak, by modelling a delayed effect using a lagged correlation, a robust negative relationship emerges. Although similar relationships have been found in older groups, our report establishes such relationships with biomarkers measured in late midlife.^3,23,24^ Given the interest in establishing valid surrogate outcomes for Alzheimer’s disease pharmaceutical research,^46^ our findings may inform the trial design in which the fitness of plasma $\mathrm{pTa}u_{217}$ for that purpose is evaluated.

### Limitations

The chief limitation of the present analysis is our small, racially homogenous sample, drawn from a cohort that is convenience- and not population-based.^27^ The complexities of bringing plasma assays into use with heterogeneous clinical populations should not be discounted. However, recent work in WHICAP suggests relatively good concordance between plasma $\mathrm{pTa}u_{217}$ and clinical status, and no evident demographic biases.^47^ In CSF and in other plasma tau biomarkers, some have observed differences in various tau isoform levels between Black and White participants after controlling for cognitive status,^48,49^ but others have not.^50^ Future directions include assaying our extensive back catalogue of plasma and expanding our existing cohort with a more diverse group of research participants.

## Conclusion

In this report, we extend previous findings of strong relationships between plasma levels of $\mathrm{pTa}u_{217}$, concurrent PET Alzheimer’s disease biomarkers and prospective cognition to a preclinical dataset. These findings have strong implications for early detection, which is prerequisite for several major goals of Alzheimer’s disease research: understanding susceptibility and resilience factors that underlie prognosis; designing better primary and secondary Alzheimer’s disease prevention trials; and determining the relative timing and impact of Alzheimer’s disease and co-occurring pathologies on cognitive decline.

## Supplementary Material

fcad057_Supplementary_Data

## Abbreviations

Aβ =: amyloid beta
AIBL =: Australian Imaging, Biomarker & Lifestyle study
CL =: centiloid
CSF =: cerebrospinal fluid
CU =: cognitively unimpaired
CV =: coefficient of variation
DVR =: distribution volume ratio
EAOA =: estimated amyloid onset age
EDTA =: ethylenediaminetetraacetic acid
GBTM-DVR =: group-based trajectory modelled distribution volume ratio at age of plasma assessment
MCI =: mild cognitive impairment
MK =: MK-6240 tau PET tracer (florquinitau)
MSD =: Meso Scale Discovery
MTL =: medial temporal lobe
NfL =: neurofilament light-chain
NPV =: negative predictive value
PACC-3 =: Preclinical Alzheimer’s cognitive composite
PiB =: Pittsburgh imaging compound B
PPV =: positive predictive value
pTau_217_ =: phosphorylated tau 217
ROC =: receiver–operator characteristic
SUVR =: standardized uptake volume ratio
WHICAP =: Washington Heights/Inwood Columbia Aging Project
WRAP =: Wisconsin Registry for Alzheimer’s Prevention
