# Supplementary material for: Plasma phosphorylated tau 217 in preclinical Alzheimer’s disease
Source: Brain Commun. 2023 Mar 6;5(2):fcad057. doi: 10.1093/braincomms/fcad057 (PMC10066514; doi:10.1093/braincomms/fcad057)
Supplement: fcad057_Supplementary_Data [file fcad057_supplementary_data.pdf]

Supplementary material

Sensitivity set 1: Starting from primary analysis set, includes 8 additional participants excluded from primary analysis: 6 due to cognitive impairment at first plasma, 1 due to missing diagnosis at first plasma, and 1 due to phosphorylated tau-217 (pTau_217) values found to be highly influential using Cook's *d*.

Sensitivity set 2: Starting from primary analysis set, excludes 6 additional observations with measured pTau_217 values below the lower limit of detection.

Supplementary Table 1

Results of an exploratory linear mixed effect model of $pTau_{217}$ as a function of the interaction between PiB positivity, years of education, and age. Model included a per-participant random intercept. $pTau_{217}$ = phosphorylated tau 217.

	Exploratory model		
Predictors	*Estimates*	*CI*	*p*
Intercept	0.23	0.20 – 0.27	**<0.001**
Education [Years, centered]	-0.00	-0.02 – 0.02	0.928
Amyloid positivity	0.19	0.14 – 0.25	**<0.001**
Age [centered], linear	0.00	-0.00 – 0.00	0.350
Education [centered] x Amyloid positivity	-0.02	-0.04 – 0.01	0.183
Education [centered] x Age [centered], linear	0.00	-0.00 – 0.00	0.818
Amyloid positivity x Age [centered], linear	0.02	0.02 – 0.03	**<0.001**
Education [centered] x Amyloid positivity x Age [centered], linear	-0.00	-0.00 – 0.00	0.504
**Random Effects**			
σ^2	0.01		
$\tau_{00\ Reggieid}$	0.03		
ICC	0.81		
$N_{Reggieid}$	165		
Observations	515		
Marginal R^2 / Conditional R^2	0.357 / 0.879		

Supplementary Table 2

Results of linear mixed effect models of PACC-3 as a function of covariates plus baseline plasma $pTau_{217}$ and its interaction with age, in two sensitivity sets. The first sensitivity set includes the primary analysis set plus eight additional participants who were cognitively impaired at first plasma draw (N=6), who did not have a consensus diagnosis at this visit (N=1) or who had extreme values of $pTau_{217}$ (N=1). The second sensitivity set includes the primary analysis set, minus 6 observations on 5 participants for which the observed $pTau_{217}$ values were below the lower limit of detection. In both models, age was modeled as a second-degree polynomial. Models included a per-participant random intercept. PACC-3 = three-test preclinical Alzheimer's cognitive composite; $pTau_{217}$ = phosphorylated tau 217.

	**Sensitivity set 1**			**Sensitivity set 2**		
Predictors	*Estimates*	*CI*	*p*	*Estimates*	*CI*	*p*
Intercept	-4.24	-5.89 – -2.60	**<0.001**	-4.14	-5.84 – -2.45	**<0.001**
Sex [Male]	-0.55	-0.83 – -0.26	**<0.001**	-0.49	-0.76 – -0.21	**0.001**
Education [Years]	0.08	0.00 – 0.15	**0.041**	0.10	0.03 – 0.18	**0.006**
Baseline literacy	0.03	0.01 – 0.04	**0.003**	0.02	0.00 – 0.04	**0.017**
Practice	0.15	0.08 – 0.21	**<0.001**	0.14	0.08 – 0.21	**<0.001**
Age [centered], linear	-0.17	-0.31 – -0.03	**0.020**	-0.05	-0.23 – 0.14	0.623
Age [centered], quadratic	-0.08	-0.11 – -0.06	**<0.001**	-0.09	-0.11 – -0.06	**<0.001**
Baseline pTau217	-0.00	-0.00 – -0.00	**0.001**	-0.00	-0.00 – -0.00	**0.027**
Baseline pTau217 x Age [centered], linear	-0.06	-0.07 – -0.04	**<0.001**	-0.08	-0.09 – -0.06	**<0.001**
Baseline pTau217 x Age [centered], quadratic	-0.00	-0.00 – -0.00	**0.049**	-0.00	-0.00 – 0.00	0.524
**Random Effects**						
σ^2	0.20			0.20		
τ_{00}	0.69 Reggieid			0.63 Reggieid		
ICC	0.77			0.76		

N	172 Reggieid	165 Reggieid
Observations	523	503
Marginal R^2 / Conditional R^2	0.345 / 0.851	0.332 / 0.842

Supplementary Table 3

Results of an exploratory linear mixed effect model of PACC-3 as a function of covariates plus baseline plasma $pTau_{217}$ and its interaction with age and education. Age was modeled as a second-degree polynomial. Model included a per-participant random intercept. PACC-3 = three-test preclinical Alzheimer's cognitive composite; $pTau_{217}$ = phosphorylated tau 217.

	**Exploratory model**		
Predictors	*Estimates*	*CI*	*p*
Intercept	-2.74	-4.69 – -0.79	**0.006**
Sex [Male]	-0.51	-0.79 – -0.23	**<0.001**
Baseline literacy	0.02	0.01 – 0.04	**0.010**
Practice	0.15	0.08 – 0.22	**<0.001**
Education [Years, centered]	0.10	0.02 – 0.17	**0.018**
Baseline pTau217	-0.05	-0.24 – 0.14	0.589
Age [centered], linear	-0.09	-0.11 – -0.06	**<0.001**
Age [centered], quadratic	-0.00	-0.00 – -0.00	**0.023**
Education [centered] x Baseline pTau217	0.03	-0.05 – 0.11	0.413
Education [centered] x Age [centered], linear	-0.01	-0.01 – 0.00	0.070
Baseline pTau217 x Age [centered], linear	-0.08	-0.10 – -0.05	**<0.001**
Education [centered] x Age [centered], quadratic	0.00	-0.00 – 0.00	0.774
Baseline pTau217 x Age [centered], quadratic	-0.00	-0.00 – 0.00	0.724
Education [centered] x Baseline pTau217 x Age [centered], linear	0.00	-0.01 – 0.01	0.507
Education [centered] x Baseline pTau217 x Age [centered], quadratic	-0.00	-0.00 – 0.00	0.433
**Random Effects**			
σ^2	0.20		
$\tau_{00\ Reggieid}$	0.64		
ICC	0.77		
$N_{Reggieid}$	165		
Observations	509		

Marginal R^2 / Conditional R^2	0.334 / 0.844

Supplementary Figure Captions

Supplementary Figure 1. Longitudinal plasma pTau$_{217}$, sensitivity set 1 (including 6 participants who were cognitively impaired at baseline; one with no baseline diagnosis; and one influential pTau$_{217}$ observation). Observations from a single participant are shown with connected edges. (A) Plasma pTau$_{217}$ as a function of age at blood draw. Color indicates the extent of tau burden as indicated on tau PET. (B) Plasma pTau$_{217}$ as a function of estimated PiB DVR at the time of plasma acquisition. Color indicates the extent of tau burden as indicated on tau PET. (C) Plasma pTau$_{217}$ as a function of age at blood draw. Color indicates amyloid PET positivity. Lines with shaded confidence bands represent slope estimates from a linear mixed effects model of pTau$_{217}$ as a function of the interaction of age and amyloid positivity. (D) Distribution of pTau$_{217}$ among PiB- and PiB+ participants. (E) Distribution of pTau$_{217}$ among MK- and MK+ participants. (F) Receiver-operator characteristic (ROC) curve relating pTau$_{217}$ to binary PiB status. Two positivity thresholds were considered for PiB: global DVR > 1.19 (red) and global DVR > 1.16 (blue). (G) ROC curve relating pTau$_{217}$ to binary MK status. Scans were marked as MK+ if tracer binding was evident in both medial temporal lobe and neocortex, and MK- otherwise. DVR = distribution volume ratio; GBTM-DVR = group-based trajectory modeled DVR from amyloid PET; MK = [^{18}F]-MK-6240 tau tracer; PiB = Pittsburgh compound B; pTau$_{217}$ = phosphorylated tau 217; ROC = receiver-operator characteristic.

Supplementary Figure 2. Longitudinal plasma pTau$_{217}$, sensitivity set 2 (excluding 6 observations from 5 participants in which the pTau$_{217}$ value fell below the lower limit of detection). Observations from a single participant are shown with connected

edges. (A) Plasma $pTau_{217}$ as a function of age at blood draw. Color indicates the extent of tau burden as indicated on tau PET. (B) Plasma $pTau_{217}$ as a function of estimated PiB DVR at the time of plasma acquisition. Color indicates the extent of tau burden as indicated on tau PET. (C) Plasma $pTau_{217}$ as a function of age at blood draw. Color indicates amyloid PET positivity. Lines with shaded confidence bands represent slope estimates from a linear mixed effects model of $pTau_{217}$ as a function of the interaction of age and amyloid positivity. (D) Distribution of $pTau_{217}$ among PiB- and PiB+ participants. (E) Distribution of $pTau_{217}$ among MK- and MK+ participants. (F) Receiver-operator characteristic (ROC) curve relating $pTau_{217}$ to binary PiB status. Two positivity thresholds were considered for PiB: global DVR > 1.19 (red) and global DVR > 1.16 (blue). (G) ROC curve relating $pTau_{217}$ to binary MK status. Scans were marked as MK+ if tracer binding was evident in both medial temporal lobe and neocortex, and MK- otherwise. DVR = distribution volume ratio; GBTM-DVR = group-based trajectory modeled DVR from amyloid PET; MK = [^{18}F]-MK-6240 tau tracer; PiB = Pittsburgh compound B; $pTau_{217}$ = phosphorylated tau 217; ROC = receiver-operator characteristic.

Supplementary Figure 3. Relationship between $pTau_{217}$ and longitudinal cognition, sensitivity set 1 (including 6 participants who were cognitively impaired at baseline; one with no baseline diagnosis; and one influential $pTau_{217}$ observation). Global cognition (PACC-3) as a function of age and baseline $pTau_{217}$ level. Low, medium, and high $pTau_{217}$ values reflect the 10th, 50th, and 90th sample percentiles.

Supplementary Figure 4. Relationship between $pTau_{217}$ and longitudinal cognition, sensitivity set 2 (excluding 6 observations from 5 participants in which the

$pTau_{217}$ value fell below the lower limit of detection). Global cognition (PACC-3) as a function of age and baseline $pTau_{217}$ level. Low, medium, and high $pTau_{217}$ values reflect the 10th, 50th, and 90th sample percentiles.

Supplementary Figure 1

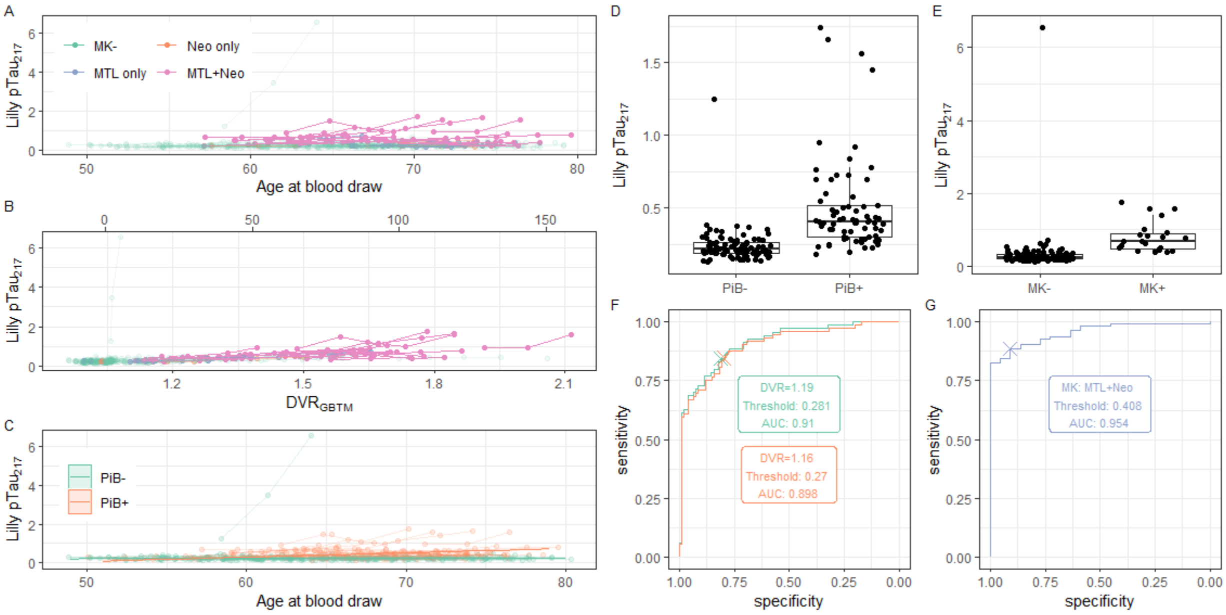

Supplementary Figure 2

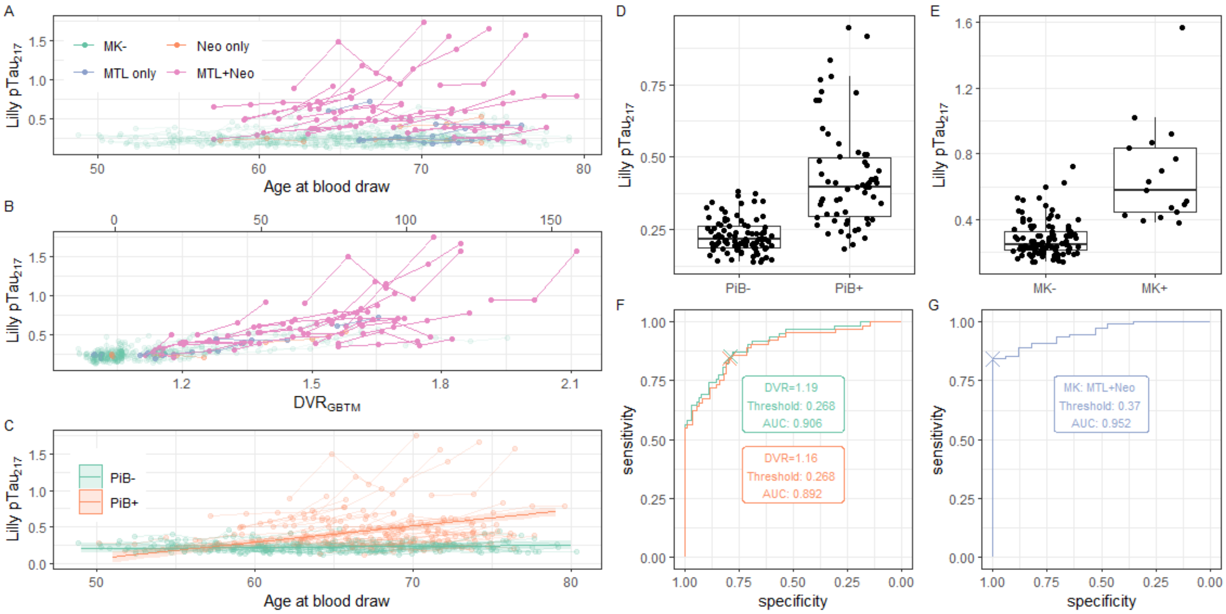

Supplementary Figure 3

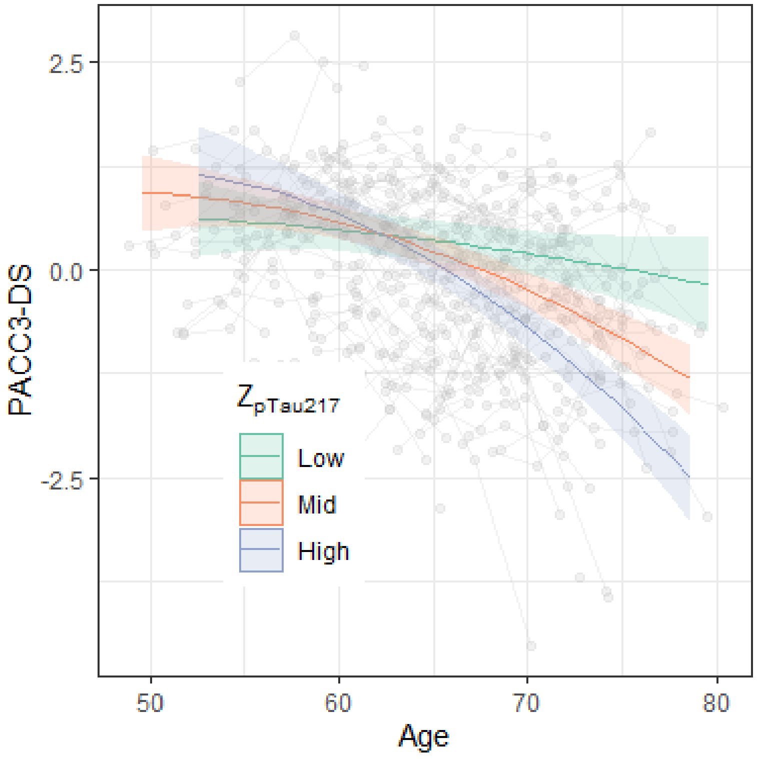

Supplementary Figure 4

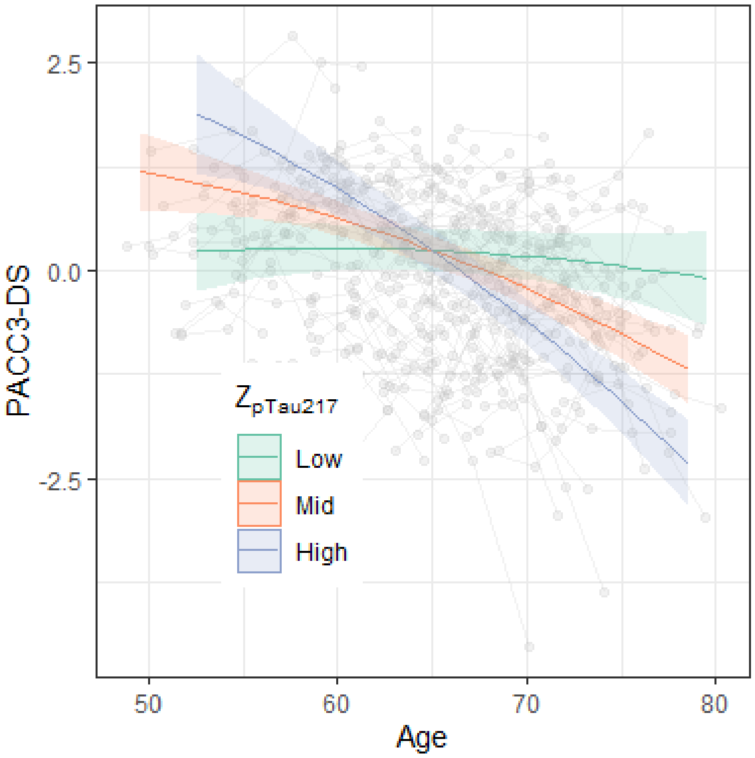

```
# Assemble analysis dataset for for Oskar Hansson's ptau217 data

# Datafile should contain
#   - Blood biomarkers at WRAP visits
#   - Cognition at the same WRAP visits
#   - Last PiB and MK
#   - Nearest PiB and MK
#   - Estimated PiB at WRAP visits (GBTM and ILLA)

library(dplyr)
library(tidyr)
library(odbc)
library(readxl)
library(lubridate)
library(emj)

# Constants for Sweden/Roche CSF data
LLMR        <- c(0,200,8,80,0,0,0,0,1.5)
names(LLMR) <-
c("ABeta_1_40","ABeta_1_42","pTau","tTau","Inv_ABeta_1_42_derived","ABeta_1_42_40_derived","pTau_ABeta_1_42_derived","tTau_ABeta_1_42_
derived","IL6")
ULMR        <- c(Inf,1700,120,1300,Inf,Inf,Inf,Inf,Inf)
names(ULMR) <-
c("ABeta_1_40","ABeta_1_42","pTau","tTau","Inv_ABeta_1_42_derived","ABeta_1_42_40_derived","pTau_ABeta_1_42_derived","tTau_ABeta_1_42_
derived","IL6")

cutoff        <- c(0.046, 0.038, 513.9, 24.8, 293, 266, 1296, 209)
names(cutoff) <- c("ABeta_1_42_40_derived","pTau_ABeta_1_42_derived", "ABeta_1_42", "pTau", "tTau", "a_Synuclein", "Neurogranin",
"NFL")


picklist <- read.csv("./data/hansson_picklist_20201201.csv")

# Merging back with WRAP data
# Updated March 2022 to use most recent freezez
livedb  <- odbc::dbConnect(odbc::odbc(), dsn="WRAP2007_Prod_DOM_64")
freeze  <- odbc::dbConnect(odbc::odbc(), dsn="Freeze 2021_May")

cc <- odbc::dbGetQuery(freeze, "SELECT WRAPNo, VisNo, consensus_dx FROM ConsensusConferenceDiagnosis")

dm <- odbc::dbGetQuery(freeze, "SELECT Demographics.WRAPNo, gender, EdYears_Coded_Max20, WRAPRaceAssignment, all1, all2
                       FROM (Demographics LEFT JOIN MiscVars ON Demographics.WRAPNo = MiscVars.WRAPNo)
                       LEFT JOIN APG ON Demographics.WRAPNo = APG.WRAPNo") %>%
      mutate(apoe4_bin = ifelse(is.na(all2), NA,
                                ifelse(all2==4, 1, 0)),
             apoe4_ct = ifelse(is.na(all1), NA,
                               ifelse(all1==4, 2,
                                      ifelse(all2==4, 1, 0))))

vv     <- odbc::dbGetQuery(livedb, "SELECT WRAPNo, VisNo, FastTime AS FastTimeRaw, Fast_HM FROM VisitVitals") %>%
          mutate(FastTime = ifelse(FastTimeRaw < 888, FastTimeRaw, NA),
```

```
                 FastImputed = ifelse(is.na(FastTime), 0, FastTime))

cdr    <- read.csv("G:/Team/AD_Program_InternalCollab/UP centiles/data/Freeze 2020_May/all_up_centiles_freeze21_20210118_wide.csv")
%>%
          filter(Data.Source=="WRAP") %>%
          select(Reggieid, VisNo=Visit_Number, cdr.xw, cdr.var, cdrsb.xw, cdrsb.var)

np     <- odbc::dbGetQuery(freeze,
                           "SELECT np.WRAPNo, np.VisNo, ttotal, drraw, wmsrtot, wmsrtot2,
                            bvmttot As bvmttot_chr, bvmtdel As bvmtdel_chr,
                            iqdspf, iqdspb, iqlnsr,
                            trla, trlb, waisrtot, StroopCW_Cor,
                            flucfl, animtotraw, bnttot, minttot,
                            mmsetot, cdtts, z_pacc3_ds, z_pacc4_ds_mmse, z_pacc4_ds_cfl
                           FROM NeuropsychScores np LEFT JOIN CompositeScores cs
                           ON np.WRAPNo=cs.WRAPNo AND np.VisNo=cs.VisNo") %>%
          mutate(bvmttot = as.numeric(bvmttot_chr),
                 bvmtdel = as.numeric(bvmtdel_chr)) %>%
          group_by(WRAPNo, VisNo) %>%
          pivot_longer(cols=c(ttotal, drraw, wmsrtot, wmsrtot2, bvmttot, bvmtdel,
                              iqdspf, iqdspb, iqlnsr,
                              trla, trlb, waisrtot, StroopCW_Cor,
                              flucfl, animtotraw, bnttot, minttot,
                              mmsetot, cdtts, z_pacc3_ds, z_pacc4_ds_mmse, z_pacc4_ds_cfl),
                       names_to="measure",
                       values_to="value") %>%
          arrange(WRAPNo, VisNo) %>%
          filter(!is.na(value) & value<888) %>%
          pivot_wider(id_cols=c("WRAPNo","VisNo"),
                      names_from="measure",
                      values_from="value",
                      values_fill=NA)

practice <- select(np, WRAPNo, VisNo, z_pacc3_ds) %>%
            filter(!is.na(z_pacc3_ds)) %>%
            arrange(WRAPNo, VisNo) %>%
            group_by(WRAPNo) %>%
            mutate(practice = (c(1:n()))-1) %>%
            ungroup()

npbase <-           odbc::dbGetQuery(freeze,
                                     "SELECT WRAPNo, VisNo, iq4fulls, readstn
                           FROM NeuropsychScores") %>%
                    pivot_longer(cols=c(iq4fulls, readstn),
                                 names_to="measure",
                                 values_to="value") %>%
                    arrange(WRAPNo, VisNo) %>%
                    filter(!is.na(value) & value<888) %>%
                    group_by(WRAPNo, measure) %>%
                    slice(1) %>%
                    pivot_wider(id_cols=c("WRAPNo"),
```

```
                                names_from="measure",
                                names_prefix="base_",
                                values_from="value",
                                values_fill=NA)

npall            <- merge(npbase, np, all=TRUE) %>%
                    merge(practice, all=TRUE)


picklist_wrapdb  <- merge(picklist, vv, all.x=TRUE) %>%
                    merge(dm, all.x=TRUE) %>%
                    merge(cc, all.x=TRUE) %>%
                    merge(npall, all.x=TRUE)


# Criterion measurements
# PiB observations

pibfile    <- "./data/panda_pib_dvr_20220316.csv"
pib.df     <- read.csv(pibfile, skip=1) %>%
  select(Reggieid=reggieid,
         pib_age=age_at_appointment, pib_index) %>%
  arrange(Reggieid, pib_age) %>%
  group_by(Reggieid) %>%
  mutate(pibno = c(1:n())) %>%
  ungroup() %>%
  mutate(pib_bin = factor(ifelse(is.na(pib_index), NA, ifelse(pib_index>1.19, "PiB+", "PiB-")),
                          levels=c("PiB-","PiB+"),
                          ordered=TRUE),
         pib_bin_lib = factor(ifelse(is.na(pib_index), NA, ifelse(pib_index>1.16, "PiB+", "PiB-")),
                              levels=c("PiB-","PiB+"),
                              ordered=TRUE),
         pib_tern = factor(ifelse(is.na(pib_index), NA,
                                  ifelse(pib_index>1.19, "PiB+",
                                         ifelse(pib_index>1.15, "PiB?", "PiB-"))),
                           levels=c("PiB-","PiB?","PiB+"),
                           ordered=TRUE),
         pib_quatern = factor(ifelse(is.na(pib_index), NA,
                                     ifelse(pib_index>1.19, "PiB+",
                                            ifelse(pib_index>1.15, "PiB?+",
                                                   ifelse(pib_index>1.1, "PiB?-", "PiB-")))),
                              levels=c("PiB-","PiB?-","PiB?+","PiB+"),
                              ordered=TRUE))
pibconv.df <- filter(pib.df, Reggieid %in% picklist_wrapdb$Reggieid) %>%
  group_by(Reggieid) %>%
  arrange(Reggieid, pib_age) %>%
  summarize(min_pib_age = first(pib_age),
            max_pib_age = last(pib_age),
            first_pib_index = first(pib_index),
            first_pib_bin = first(pib_bin),
            first_pib_tern = first(pib_tern),
```

```
            first_pib_quatern = first(pib_quatern),
            last_pib_index = last(pib_index),
            last_pib_bin = last(pib_bin),
            last_pib_tern = last(pib_tern),
            last_pib_quatern = last(pib_quatern),
            max_pib_index = max(pib_index),
            max_pib_bin = max(pib_bin),
            max_pib_bin_lib = max(pib_bin_lib),
            max_pib_tern = max(pib_tern),
            max_pib_quatern = max(pib_quatern),
            convert_pib = if (min_pib_age==max_pib_age) { NA }
                          else { (first_pib_index < 1.19 & last_pib_index > 1.19) },
            n_neg_pib = length(pib_bin[pib_index <= 1.1]),
            n_pib = n())


# Finding closest PiB measurement
# Don't necessarily want just closest EDTA. Want whole swath of them
picklist_nearest_pib  <- merge(pib.df, pibconv.df, by="Reggieid") %>%
                         arrange(Reggieid, pib_age) %>%
                         merge(picklist_wrapdb, by="Reggieid") %>%
                         mutate(delta_pib_lab = abs(pib_age - AgeAtBlood)) %>%
                         group_by(Reggieid, pib_age) %>%
                         arrange(Reggieid, delta_pib_lab) %>%
                         slice(1) %>%
                         ungroup() %>%
                         select(Reggieid, pib_age, AgeAtBlood, delta_pib_lab, VisNo, pib_index, pib_bin, pib_bin_lib,
                                max_pib_bin, max_pib_bin_lib) %>%
                         arrange(Reggieid, desc(pib_bin), delta_pib_lab) %>%
                         group_by(Reggieid) %>%
                         slice(1)

picklist_nearest_pib_1row_2y <- filter(picklist_nearest_pib, delta_pib_lab<2)
write.csv(picklist_nearest_pib_1row_2y, file="./data/ptau217_merged_covariates_for_roc.csv", row.names=FALSE)


gbtm_file <- "G:/Team/AD_Program_InternalCollab/AmyloidModeling_ADNI-WRAP/data/est_dvr_at_edta_gbtm_pcws_updtd.xlsx"
# Centiloid formula per email from SCJ 2022-03-24 2:06 pm
gbtm_est <- read_excel(path=gbtm_file) %>%
            select(Reggieid=reggieid, AgeAtBlood, gbtm_pred = pred_pibdvr_at_edta, gbtm_chron = edta_chron) %>%
            mutate(gbtm_chron_trunc = ifelse(gbtm_chron<=-10, -10, gbtm_chron),
                   gbtm_centiloid = gbtm_pred*148.33-154.96)

illa_file <- "./data/illa-estimates_at_edta_ages.csv"
illa_est <- read.csv(file=illa_file) %>%
            select(Reggieid=reggieid, AgeAtBlood=age, illa_pred=estval)

picklist_est_pib <- merge(picklist_wrapdb,
                          pibconv.df, all.x=TRUE) %>%
                    merge(gbtm_est, all.x=TRUE) %>%
                    merge(illa_est, all.x=TRUE)
```

```
# MK
factor_yn <- function(x) { return(factor(x, levels=c("N","Y"), ordered=TRUE))}
mkfile     <- "./data/panda_mk_braak_20220316.csv"
mk.df      <- read.csv(mkfile, skip=1) %>%
  select(Reggieid=reggieid,
         mk_age=Pet.age_at_appointment, braak_1:braak_6) %>%
  mutate(across(.cols=braak_1:braak_6, .fns=factor_yn)) %>%
  mutate(mtl_bin = pmax(braak_1, braak_2, na.rm=TRUE),
         neo_bin = pmax(braak_3, braak_4, braak_5, braak_6, na.rm=TRUE),
         mk_cat = factor(ifelse(is.na(mtl_bin), NA,
                          ifelse(mtl_bin=='N',
                            ifelse(neo_bin=='N', 'MK-', 'Neo only'),
                            ifelse(neo_bin=='N', 'MTL only', 'MTL+Neo'))),
                         levels=c('MK-', 'Neo only', 'MTL only', 'MTL+Neo'),
                         ordered=TRUE))
mkconv.df <- filter(mk.df, Reggieid %in% picklist_wrapdb$Reggieid) %>%
  group_by(Reggieid) %>%
  arrange(Reggieid, mk_age) %>%
  summarize(min_mk_age = first(mk_age),
            max_mk_age = last(mk_age),
            first_mk_cat = first(mk_cat),
            last_mk_cat = last(mk_cat),
            min_mk_cat = min(mk_cat, na.rm=TRUE),
            max_mk_cat = max(mk_cat, na.rm=TRUE),
            convert_mk = if (min_mk_age==max_mk_age) { NA }
                         else { (min_mk_cat < max_mk_cat) })

picklist_nearest_mk  <- merge(mk.df, mkconv.df, by="Reggieid") %>%
  arrange(Reggieid, mk_age) %>%
  merge(picklist_wrapdb, by="Reggieid") %>%
  mutate(delta_mk_lab = abs(mk_age - AgeAtBlood)) %>%
  group_by(Reggieid, mk_age) %>%
  arrange(Reggieid, delta_mk_lab) %>%
  slice(1) %>%
  ungroup() %>%
  select(Reggieid, mk_age, AgeAtBlood, delta_mk_lab, VisNo, mk_cat) %>%
  mutate(mk_bin = factor(ifelse(is.na(mk_cat), NA, ifelse(mk_cat=="MTL+Neo", 1, 0)),
                         levels=c(0,1),
                         labels=c("None or MTL/Neo only", "MTL + Neocortex"))) %>%
  arrange(Reggieid, desc(mk_bin), delta_mk_lab) %>%
  group_by(Reggieid) %>%
  slice(1)

picklist_nearest_mk_1row_2y <- filter(picklist_nearest_mk, delta_mk_lab<2)
write.csv(picklist_nearest_mk_1row_2y, file="./data/ptau217_merged_covariates_for_roc_mk.csv", row.names=FALSE)

picklist_est_pib_mk <- merge(picklist_est_pib, mkconv.df, all.x=TRUE)
```

```
# Pulling in LP information
lpfile     <- "G:/Team/Van Hulle 4-3-2019/NTK/raw data/shared_csf_panda_batch5678_20200226.csv"
lp.df      <- read.csv(lpfile, stringsAsFactors=FALSE, skip=1) %>%
  rename(Reggieid = reggieid,
         WRAPNo = wrapnum,
         ABeta_1_40_result_ng = ABeta_1_40_result,
         lp_age = age_at_appointment) %>%
  filter(Reggieid %in% picklist_wrapdb$Reggieid) %>%
  mutate(ABeta_1_40_result = nano_to_pico(ABeta_1_40_result_ng)) %>%
  arrange(Reggieid, lumbarpuncture_date_date, lp_age) %>%
  select(Reggieid, everything()) %>%
  group_by(Reggieid) %>%
  arrange(Reggieid, lp_age) %>%
  mutate(LP_Number = c(1:n()),
         lp_age = ifelse(Reggieid==8846 & lp_age==56.54, 56.52,
                         ifelse(Reggieid==5860 & lp_age==64.88, 64.91, lp_age))) %>%
  ungroup() %>%
  group_by(Reggieid, LP_Number, lp_age) %>%
  select(Reggieid, LP_Number, lp_age, ABeta_1_42_result, ABeta_1_40_result, pTau_result, NFL_result) %>%
  gather(key="assay", value="result_char", ABeta_1_42_result:NFL_result) %>%
  ungroup() %>%
  mutate(assay=gsub("_result","",assay,fixed=TRUE),
         result=ifelse(grepl("<", result_char, fixed=TRUE), LLMR[assay],
                       ifelse(grepl(">", result_char, fixed=TRUE), ULMR[assay],
                              as.numeric(result_char)))) %>%
  select(-result_char) %>%
  filter(!is.na(result)) %>% # Remove NA rows - Reggieid 1681 dup
  group_by(Reggieid, assay, lp_age) %>%
  arrange(Reggieid, assay, lp_age, result) %>%
  spread(key="assay", value="result", fill=NA) %>%
  ungroup() %>%
  group_by(Reggieid, LP_Number) %>%
  mutate(ABeta_1_42_40_derived = ifelse(is.na(ABeta_1_42) | is.na(ABeta_1_40), NA, golden_round(ABeta_1_42/ABeta_1_40)),
         pTau_ABeta_1_42_derived = ifelse(is.na(pTau) | is.na(ABeta_1_42), NA, golden_round(pTau/ABeta_1_42)),
         ABeta_1_42_40_derived_bin = ABeta_1_42_40_derived<cutoff["ABeta_1_42_40_derived"],
         pTau_ABeta_1_42_derived_bin = pTau_ABeta_1_42_derived>cutoff["pTau_ABeta_1_42_derived"],
         ABeta_1_42_bin = ABeta_1_42<cutoff["ABeta_1_42"],
         pTau_bin = pTau>cutoff["pTau"])  %>%
  ungroup()
lpconv.df  <- group_by(lp.df, Reggieid) %>%
  filter(!is.na(ABeta_1_42_40_derived) & !is.na(pTau_ABeta_1_42_derived)) %>%
  arrange(Reggieid, lp_age) %>%
  summarize(first_lp_age = first(lp_age),
            last_lp_age = last(lp_age),
            first_ABeta_1_42_40_derived_bin = first(ABeta_1_42_40_derived_bin),
            last_ABeta_1_42_40_derived_bin = last(ABeta_1_42_40_derived_bin),
            max_ABeta_1_42_40_derived_bin = max(ABeta_1_42_40_derived_bin),
            first_pTau_ABeta_1_42_derived_bin = first(pTau_ABeta_1_42_derived_bin),
```

```
            last_pTau_ABeta_1_42_derived_bin = last(pTau_ABeta_1_42_derived_bin),
            max_pTau_ABeta_1_42_derived_bin = max(pTau_ABeta_1_42_derived_bin),
            convert_ABeta_1_42_40_derived = if (first_lp_age==last_lp_age) { NA }
                                            else {(first_ABeta_1_42_40_derived_bin==0 &
                                                   last_ABeta_1_42_40_derived_bin==1)},
            convert_pTau_ABeta_1_42_derived = if (first_lp_age==last_lp_age) { NA }
                                              else {(first_pTau_ABeta_1_42_derived_bin==0 &
                                                     last_pTau_ABeta_1_42_derived_bin==1)})



picklist_est_pib_mk_lp <- merge(picklist_est_pib_mk, lpconv.df, all.x=TRUE)


write.csv(picklist_est_pib_mk_lp, file="./data/ptau217_merged_covariates_for_analyses_v2.csv", row.names=FALSE)


# Checking changes
old_covs <- read.csv("./data/ptau217_merged_covariates_for_analyses.csv")
new_covs <- picklist_est_pib_mk_lp
check_covs <- merge(select(old_covs, Reggieid, VisNo, old_age=AgeAtBlood, old_max_pib=max_pib_bin, old_max_mk=max_mk_bin),
                    select(new_covs, Reggieid, VisNo, new_age=AgeAtBlood, new_max_pib=max_pib_bin, new_max_mk=max_mk_cat),
                    all=TRUE)

# Changed max_pib_bin: just 3
group_by(check_covs, old_max_pib, new_max_pib) %>% summarize(N=n())

# Altered max_mk_bin: All, since we modified our strategy.
group_by(check_covs, old_max_mk, new_max_mk) %>% summarize(N=n()) %>% arrange(desc(N))

# Nobody appears newly here (that makes sense; I think subjects needed to have scans to be included
```
